# Ameliorative Effects of Exogenous Potassium Nitrate on Antioxidant Defense System and Mineral Nutrient Uptake in Radish (*Raphanus sativus* L.) under Salinity Stress

**DOI:** 10.1021/acsomega.3c01039

**Published:** 2023-06-12

**Authors:** Amany H. A. Abeed, Muhammad Hamzah Saleem, Muhammad Ahsan Asghar, Sahar Mumtaz, Amina Ameer, Baber Ali, Mona S. Alwahibi, Mohamed S. Elshikh, Sezai Ercisli, Mohsen Mohamed Elsharkawy, Shafaqat Ali, Fathia A. Soudy

**Affiliations:** † Department of Botany and Microbiology, Faculty of Science, Assiut University, Assiut 71516, Egypt; ‡ Office of Academic Research, Office of VP for Research & Graduate Studies, Qatar University, Doha 2713, Qatar; § Department of Biological Resources, Agricultural Institute, Centre for Agricultural Research, ELKH, Brunszvik U. 2, 2462 Martonvásár, Hungary; ∥ Department of Botany, Division of Science and Technology, University of Education, Lahore 54770, Pakistan; ⊥ Department of Botany, University of Agriculture, Faisalabad 38000, Pakistan; # Department of Plant Sciences, Quaid-i-Azam University, Islamabad 45320, Pakistan; ∇ Department of Botany and Microbiology, College of Science, King Saud University, Riyadh 11451, Saudi Arabia; ○ Department of Horticulture Faculty of Agriculture, Ataturk University, Erzurum 25240, Türkiye; ◆ HGF Agro, Ata Teknokent, TR-25240 Erzurum, Türkiye; ¶ Department of Agricultural Botany, Faculty of Agriculture, Kafrelsheikh University, Kafr el-Sheikh 33516, Egypt; ⋈ Department of Environmental Sciences and Engineering, Government College University Faisalabad, Faisalabad 38000, Pakistan; ⧓ Department of Biological Sciences and Technology, China Medical University, Taichung City 40402, Taiwan; ⧖ Genetics and Genetic Engineering Department, Faculty of Agriculture, Benha University, Moshtohor 13736, Egypt

## Abstract

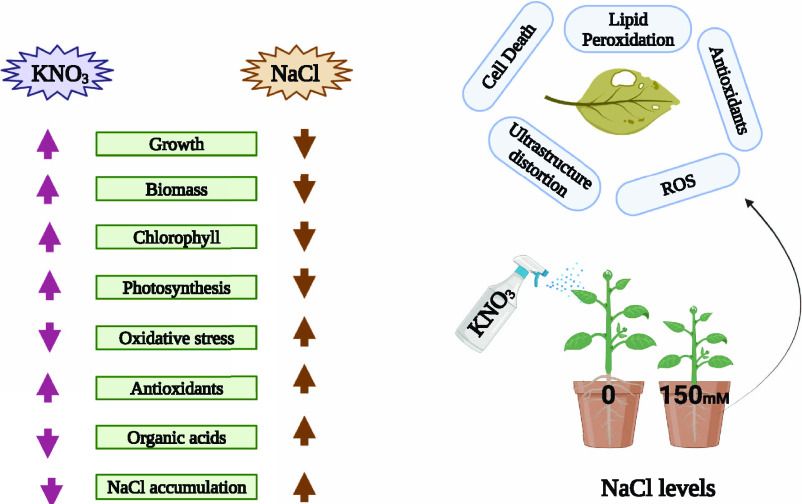

Soil salinization has become a major issue around the world in recent years, as it is one of the consequences of climate change as sea levels rise. It is crucial to lessen the severe consequences of soil salinization on plants. A pot experiment was conducted to regulate the physiological and biochemical mechanisms in order to evaluate the ameliorative effects of potassium nitrate (KNO_3_) on *Raphanus sativus* L. genotypes under salt stress. The results from the present study illustrated that the salinity stress induced a significant decrease in shoot length, root length, shoot fresh weight, shoot dry weight, root fresh weight, root dry weight, number of leaves per plant, leaf area chlorophyll-a, chlorophyll-b, total chlorophyll, carotenoid, net photosynthesis, stomatal conductance, and transpiration rate by 43, 67, 41, 21, 34, 28, 74, 91, 50, 41, 24, 34, 14, 26, and 67%, respectively, in a 40 day radish while decreased by 34, 61, 49, 19, 31, 27, 70, 81, 41, 16, 31, 11, 21, and 62%, respectively, in Mino radish. Furthermore, MDA, H_2_O_2_ initiation, and EL (%) of two varieties (40 day radish and Mino radish) of *R. sativus* increased significantly (*P* < 0.05) by 86, 26, and 72%, respectively, in the roots and also increased by 76, 106, and 38% in the leaves in a 40 day radish, compared to the untreated plants. The results also elucidated that the contents of phenolic, flavonoids, ascorbic acid, and anthocyanin in the two varieties (40 day radish and Mino radish) of *R. sativus* increased with the exogenous application of KNO_3_ by 41, 43, 24, and 37%, respectively, in the 40 day radish grown under the controlled treatments. Results indicated that implementing KNO_3_ exogenously in the soil increased the activities of antioxidants like SOD, CAT, POD, and APX by 64, 24, 36, and 84% in the roots and also increased by 21, 12, 23, and 60% in the leaves of 40 day radish while also increased by 42, 13, 18, and 60% in the roots and also increased by 13, 14, 16, and 41% in the leaves in Mino radish, respectively, in comparison to those plants grown without KNO_3_. We found that KNO_3_ substantially improved plant growth by lowering the levels of oxidative stress biomarkers, thereby further stimulating the antioxidant potential system, which led to an improved nutritional profile of both *R. sativus* L. genotypes under normal and stressed conditions. The current study would offer a deep theoretical foundation for clarifying the physiological and biochemical mechanisms by which the KNO_3_ improves salt tolerance in *R. sativus* L. genotypes.

## 1 Introduction

Reduction in soil productivity owing to salt accumulation in rhizosphere is one of the most common phenomena occurring worldwide.^1,2^ Salinity is extremely detrimental for plants and imperils almost 20% of cultivated lands, which occupies 6% of the total world.^3^ Saline soils are characterized by the accumulation of excessive salts, which impart injurious effects on plant growth by reducing osmotic potential, interrupting ionic uptake, nutrient disparities, and ionic toxicity.^4−6^ Elevated levels of salt in soil cause drastic consequences in plant like reduced gemination, decreased seedling growth, and inadequate flowering and fruiting, which results in poor quality and declined crop yield.^7^ Plant development is hampered by salt stress due to osmotic and ionic effects that disturb ion balance.^8,9^ Another study revealed that the indirect impacts of salt stress, including oxidative stress and reduced photosynthesis, have been linked to growth yield loss in saline conditions.^10^ Osmotic stress induced by the salt causes ABA biosynthesis and, as a result, stomatal closure.^11^ Salt may build in the apoplast, cytoplasm, or chloroplast under extreme salt stress, affecting photosynthetic metabolism directly.^12,13^

By enhancing translocation and preserving water balance, potassium reduces the negative effects of salt stress. K^+^ participates in numerous crucial roles in many physiological and biochemical processes, including stomatal closure, signal transduction, protein synthesis, photosynthesis, electrolyte balance, phloem filling, and the evacuation of excessive free radicals.^14,15^ K facilitates the movement of metabolites and inorganic anions in cells as well as the regulation of cytoplasmic pH.^14^ Plants cultivated in K-deficient agricultural soils can use a variety of strategies to keep the optimal required amount of K, including the increased potential to uptake K^+^ from soil, potassium ion redistribution between cytosolic and vacuolar pools, cytosolic homeostasis, and cytosolic alterations.^16^

At the initial stages of plant development, the root system may not develop sufficiently to acquire sufficient nutrients from the soil; in this situation, foliar fertilizer spray could be used to provide vital nutrients such as potassium (K+) and phosphorus (P+) to the plants.^17^ A high NaCl content in the soil causes P+ and K+ deficits in tomato^18^ and cucumber.^19^ Fertilizing plants with K+ to increase the K+/Na+ ratio is an efficient method of enhancing plant tolerance to salt stress.^20^ Radish (*Raphanus sativus* L.) belongs to the Brassicaceae family and is either tolerable or fairly sensitive to salt. The tap root of radishes has been consumed worldwide in the form of pickles, salads, and curries due to their high nutritional values. Apart from the roots, leaves and sprouts have also been reported to have nutritional and medicinal importance. The extracts of radishes have been employed to treat stomach disorders, constipation, urinary infections, hepatic inflammation, cardiac disorders, and ulcers in folk medicine since ancient times. The current study aimed to determine whether KNO_3_ could effectively reduce the detrimental effects of salinity on *R. sativus* L., in addition, the extent to which the foliar application of KNO_3_ contributes to the oxidative equilibrium, photosynthetic signal transduction, and ion homeostasis in regulating salt resistance.

## 2 Materials and Methods

### 2.1 Experimental Setup

Two varieties (Mino and 40 day) of radish (*Raphanus sativus* L.) were selected for this experiment and attained from Ayyub Agricultural Research Institute (AARI), Faisalabad. This research was established in the Department of Botany, Government College University, Faisalabad 38000, Punjab, Pakistan (31°24/N, 73°04/E), by employing pots containing sand (10 kg). Almost 10 seeds of radish were sown in each plastic pot. Following the germination of *R. sativus* seeds, these seedlings were supplied with canal water for irrigation in control pots. The total duration of experimental treatments was 2 months under controlled conditions where they received natural light with a day/night temperature of 35/40 °C and a day/night humidity of 60/70%. Irrigation with free water and other intercultural operations was performed when needed. For the application of salt stress, 150 mM of NaCl was supplied after 14 days of germination. 0, 10, and 20 mM of potassium nitrate were applied to control and salt-stressed seedlings. Different levels of potassium nitrate was used in this study was followed by.^21^

After 2–4 weeks of potassium nitrate treatment, leaves and roots of radish were sampled for further analysis. All of the biochemical and physiological characteristics were measured by employing different techniques. The study was designed in complete randomized design (CRD) with three replications.

### 2.2 Analysis of Samples and Data Collection

Soil physicochemical characteristics were analyzed by laboratories of the Ayub Agriculture Research Institute (AARI). A measuring scale was employed to measure the shoot and root length of each radish plant. For the study of root and shoot fresh weight, weight balance was used after washing the radish plants. Radish plants were oven-dried for 48–72 h to assess the root and shoot dry weight. For dried samples, a microbalance was used to note the root and shoot dry weight.

The earlier established method was employed to analyze the chlorophyll contents of radish.^22^ IRGA was used to study the gas exchange parameters of radish plants during hot sunny days. The proline content was calculated using the method described by Bates et al.^23^ Each sample’s frozen leaf tissues (200–220 mg) were crushed and homogenized with 3% of 4 mL aqueous sulfo-salicylic acid. The extract was centrifuged at 4 °C for 15 min at 10,000*g* (Eppendorf 5804R, Germany). Glass tubes were heated in a water bath at 95 °C for 1 h after adding 2 mL of acid ninhydrin and 2 mL of glacial acetic acid to 2 mL of supernatant. To cool the reaction tubes, they were placed in an ice bath. The reaction mixture was then given 4 mL of toluene. At room temperature, the reaction tubes were stirred continuously for 15–20 s before being left undisturbed for about 30 min and their absorbance at 520 nm was measured with a spectrophotometer (Hitachi, 220, Japan).

#### 2.2.1 Total Soluble Sugars

A modified method of Irigoyen et al.^24^ was used to determine the total soluble sugar content in the ethanol-soluble fractions. The sample was vacuum-dried and dissolved in 0.1 mL of deionized water before being deproteinized with 0.1 mL of 0.3 N Ba(OH)_2_ and 0.1 mL of 5% ZnSO_4_. After centrifugation at 23,000*g* for 5 min, 0.1 mL of supernatant was reacted at 100 C for 10 min with 0.4 mL of freshly prepared anthrone reagent (100 mg anthrone + 50 mL 95% H_2_SO_4_). The total soluble sugar content was determined using a spectrophotometer at 620 nm after cooling on ice.

Following the protocol of Mukherjee and Choudhuri,^25^ a leaf sample (0.25 g) was mixed with a 5 mL solution of 6% trichloroacetic acid (TCA) and then filtered. The filtrate (2 mL) was combined with 1.0 mL of 2% 2,4 dinitrophenylhydrazine (2,4-DNPH) and one drop of 10% thiourea. After 15 min in a 100 °C water bath, the samples were cooled to room temperature and 2.5 mL of 80% H_2_SO_4_ was added to each. Using a spectrophotometer, the optical density of each sample was measured at 530 nm.

#### 2.2.2 Quantification of Antioxidant Enzymes and Oxidative Burst

Methods of Aebi,^26^ Sakharov and Ardila,^27^ and Rehman et al.^28^ were followed for the quantification of APX, POD, and SOD, respectively. The method of Kruse et al.^29^ was followed to analyze the quantity of different nutrients in radish plants.

To measure the contents of AsA and total ascorbate, the procedure illustrated by Griffith^30^ was used. Total ascorbate was determined after incubating the sample in dithiothreitol for 15 min. The DHA content was calculated by subtracting total ascorbate from reduced AsA. To determine the levels of total ascorbate, AsA, and DHA, a calibration curve prepared with AsA and DHA was used. The ratio of the AsA content to DHA content was defined as AsA/DHA.

The Nakano and Asada^31^ procedure was used to analyze CAT activity. The CAT activity was measured by adding 1.9 mL of potassium phosphate buffer (50 mM, pH 7.8), 100 mL of sample, and 1 mL of 5.9 mM H_2_O_2_ to a quartz cuvette and measuring the change in OD at 240 nm every 20 s for 2 min. To measure the contents of AsA and total ascorbate, the procedure illustrated by Aebi^26^ was used. Total ascorbate was determined after incubating the sample in dithiothreitol for 15 min. The DHA content was calculated by subtracting total ascorbate from reduced AsA. To determine the levels of total ascorbate, AsA, and DHA, a calibration curve prepared with AsA and DHA was used. The ratio of the AsA content to DHA content was defined as AsA/DHA.

For the MDA content, the leaf sample (0.25 g) was crushed in 5 mL of 5% TCA according to the procedure outlined by Heath and Packer,^32^ centrifuged, and 500 μL of the supernatant was mixed with 2 mL of 0.5% thiobarbituric acid (TBA). The mixtures were heated for 50 min in a water bath set at 95 °C, cooled in an ice bath, and then the absorbance at 600 and 532 nm was measured.

According to the Dionisio-Sese and Tobita^33^ protocol, the H_2_O_2_ content was measured colorimetrically. By homogenizing leaf tissue in phosphate buffer (50 mM, pH 6.5) containing 1 mM of hydroxylamine, H_2_O_2_ was extracted and the homogenate was centrifuged at 6000*g* for 25 min. The extracted solution was combined with 0.1% (v/v) titanium chloride in 20% (v/v) H_2_SO_4_ to measure the H_2_O_2_ content. The mixture was then centrifuged for 25 min at 6000*g*. At 410 nm, the absorbance was measured.

100 mg of fresh leaf samples were cut into 5 mm lengths and put in test tubes with 10 mL of distilled deionized water to determine electrolyte leakage. The tubes were placed in a water bath that was kept at a constant temperature of 32 °C and covered with plastic caps. An electrical conductivity meter was used to measure the medium’s initial electrical conductivity (EC_1_) after 2 h (CM-115, Kyoto Electronics, Kyoto, Japan). To completely kill the tissues and release all electrolytes, the samples were autoclaved at 121 °C for 20 min. After samples had reached a temperature of 25 °C, the final electrical conductivity (EC_2_) was assessed. The formula EL = EC_1_/EC_2_ 100 was used to express the electrolyte leakage (EL).

### 2.3 Statistical Analysis

Data regarding different biochemical and physiological attributes of radish plants were compiled by employing Statistix 8.1 software. For this experimentation, each treatment was recorded in four replicates by using 2-way ANOVA at *P* < 0.05 at HSD. The graphical presentation was carried out by using Origin-Pro 2017. The Pearson correlation coefficients between the measured variables of *R. sativus* were also calculated using the Rstudio software.

## 3 Results

### 3.1 Plant Growth Parameters

**Figure 1:**
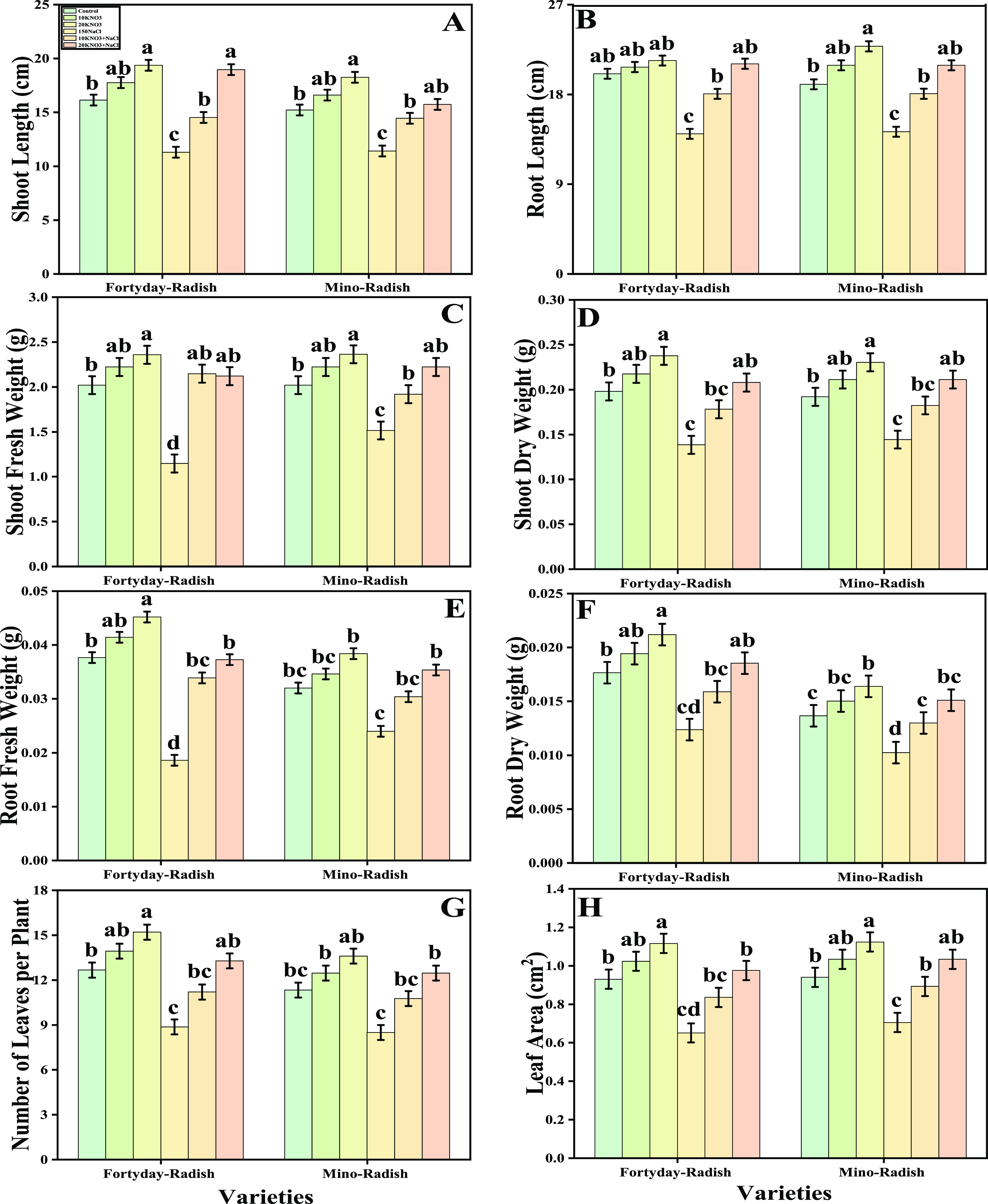
Effect of exogenous potassium nitrate (10 and 20 mM) on shoot length (A), root length (B), shoot fresh weight (C), shoot dry weight (D), root fresh weight (E), root dry weight (F), number of leaves per plant (G), and leaf area (H) on *R. sativus* grown under salt stress (150 μM). Bars sharing different letter(s) for each parameter are significantly different from each other according to Duncan’s multiple range test (*P* < 0.05). All of the data represented are the average of four replications (*n* = 4). Error bars represent the standard deviation (SD) of four replicates.

**Figure 2:**
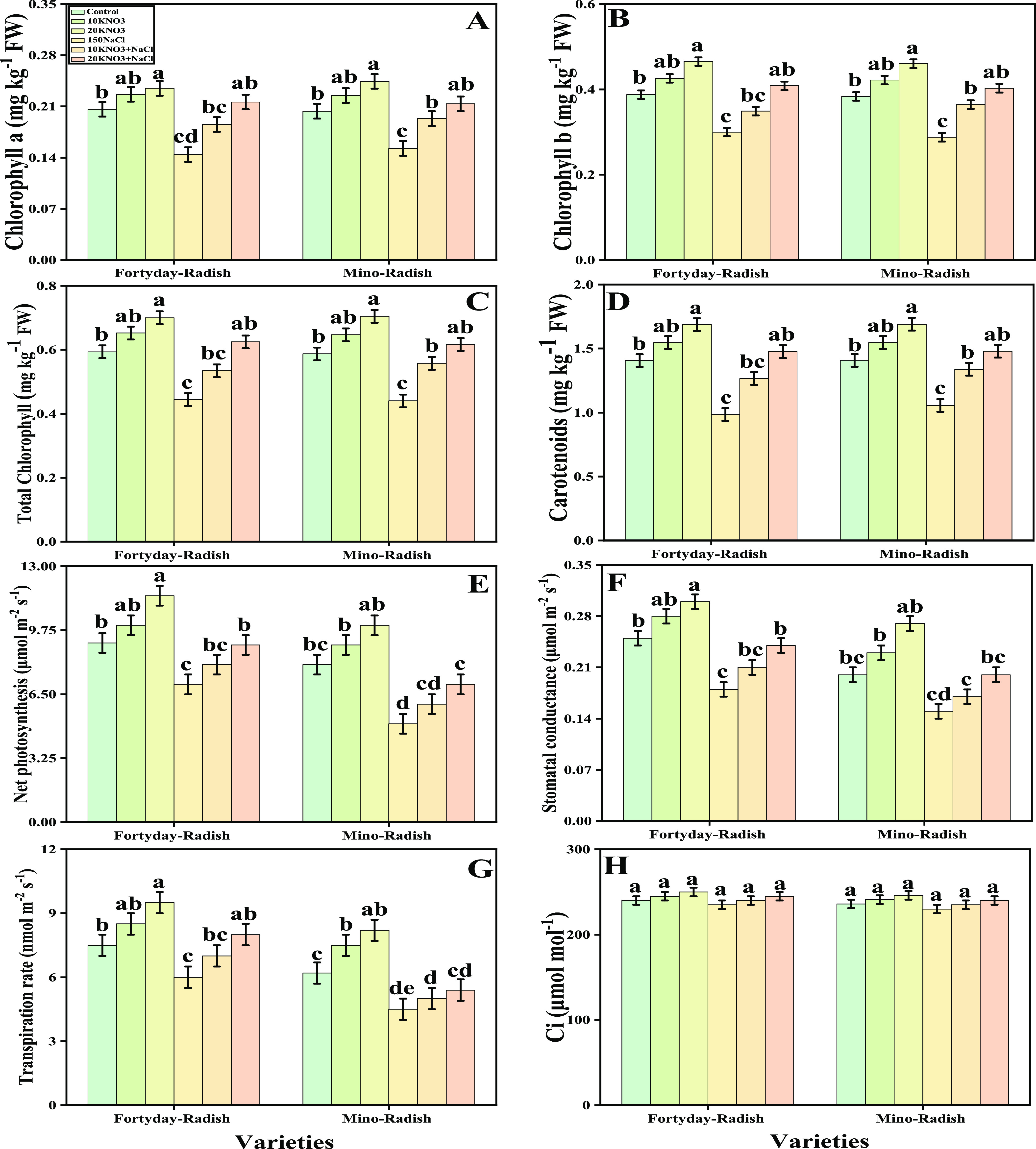
Effect of exogenous potassium nitrate (10 and 20 mM) on chlorophyll-a (A), chlorophyll-b (B), total chlorophyll (C), carotenoid (D), net photosynthesis, (E) stomatal conductance (F), transpiration rate (G), and intercellular CO_2_ (H) of *R. sativus* grown under salt stress (150 μM). Bars sharing different letter(s) for each parameter are significantly different from each other according to Duncan’s multiple range test (*P* < 0.05). All of the data represented are the average of four replications (*n* = 4). Error bars represent the standard deviation (SD) of four replicates.

In the present study, we have measured various growth and photosynthetic parameters of *R. sativus* cultivars (40 day radish and Mino radish) grown under the salinity stress with or without the application of KNO_3_. The data regarding the shoot length, root length, shoot fresh weight, shoot dry weight, root fresh weight, root dry weight, number of leaves per plant, leaf area chlorophyll-a, chlorophyll-b, total chlorophyll, carotenoid, net photosynthesis, stomatal conductance, transpiration rate, and intercellular CO_2_ are presented in Figures 1 and 2. In comparison to control, the plants grown under the treatment of 150 μM of salinity concentration in the soil showed a decrease in shoot length, root length, shoot fresh weight, shoot dry weight, root fresh weight, root dry weight, number of leaves per plant, leaf area chlorophyll-a, chlorophyll-b, total chlorophyll, carotenoid, net photosynthesis, stomatal conductance, and transpiration rate by 43, 67, 41, 21, 34, 28, 74, 91, 50, 41, 24, 34, 14, 26, and 67%, respectively, in 40 day radish while decreased by 34, 61, 49, 19, 31, 27, 70, 81, 41, 16, 31, 11, 21, and 62%, respectively, in Mino radish. Although, treatment with KNO_3_ in the soil which was not spiked with NaCl and also in the soil which was spiked with NaCl, increased significantly (*P* < 0.05) the growth and photosynthetic pigments such as shoot length, root length, shoot fresh weight, shoot dry weight, root fresh weight, root dry weight, number of leaves per plant, leaf area chlorophyll-a, chlorophyll-b, total chlorophyll, carotenoid, net photosynthesis, stomatal conductance, and transpiration rate compared to the plants which were not treated with the KNO_3_. However, results also showed that the intercellular CO_2_ showed nonsignificant results under the treatment of salinity stress in the soil with or without the application of KNO_3_.

### 3.2 Oxidative Stress Indicators

**Figure 3:**
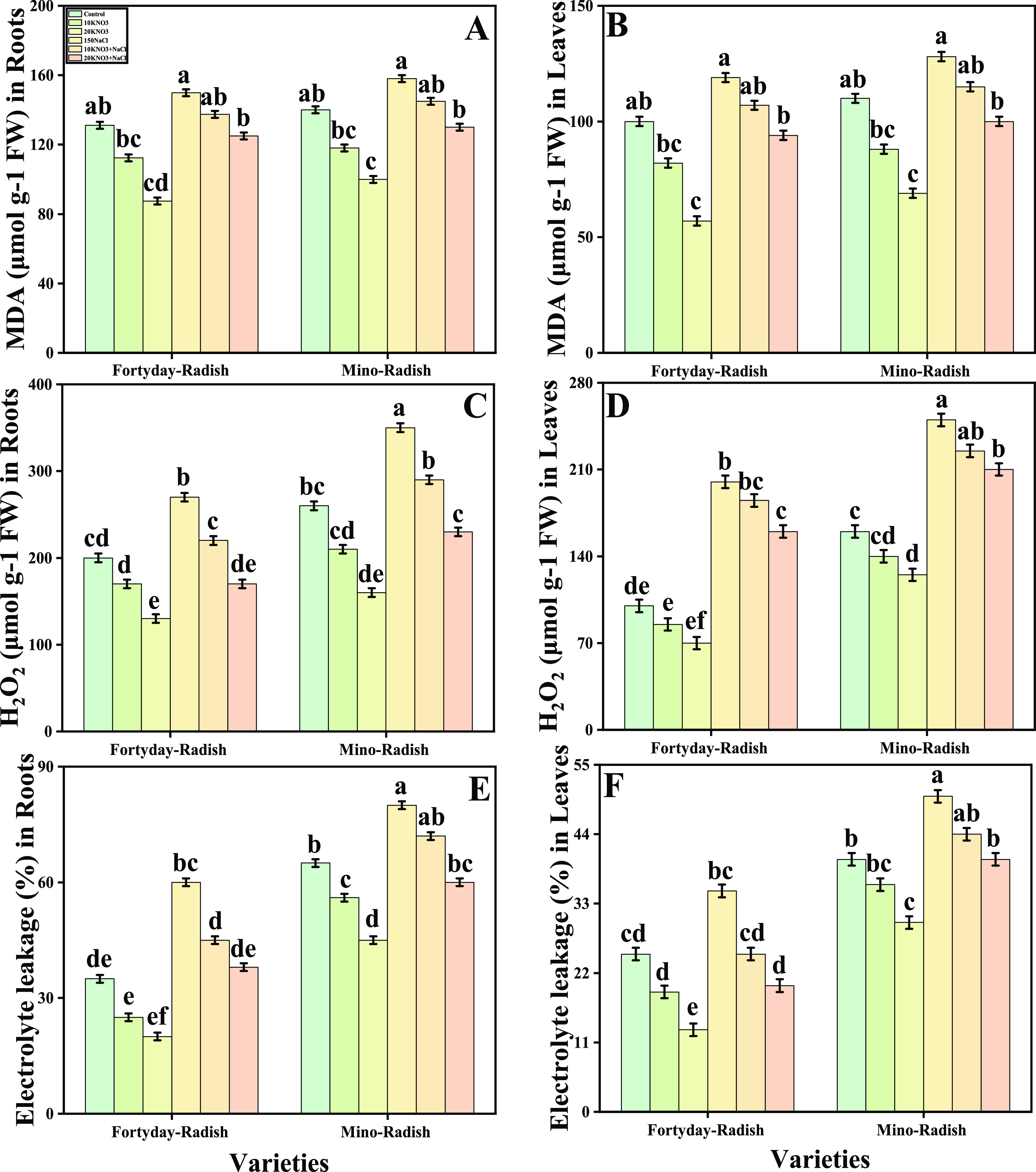
Effect of exogenous potassium nitrate (10 and 20 mM) on MDA levels in roots (A), MDA levels in shoots (B), H_2_O_2_ levels in roots (C), H_2_O_2_ levels in shoots (D), relative electrolyte leakage in roots (E), and relative electrolyte leakage in shoots (F) of *R. sativus* grown under salt stress (150 μM). Bars sharing different letter(s) for each parameter are significantly different from each other according to Duncan’s multiple range test (*P* < 0.05). All of the data represented are the average of four replications (*n* = 4). Error bars represent the standard deviation (SD) of four replicates.

In Figure 3, we have presented the oxidate stress indicators of two varieties (40 day radish and Mino radish) of *R. sativus* by the exogenous application of various levels of KNO_3_ cultivated under salt stress. Free radicals as stress signaling molecules such as H_2_O_2_, MDA, and EL in both leaves and roots of the studied plants were measured. The graphical representation was related to H_2_O_2_, MDA, and EL in plant parts of two cultivars (40 day radish and Mino radish) of *R. sativus* grown under salt stress, elucidating that the contents of MDA, H_2_O_2_ initiation, and EL (%)of two varieties (40 day radish and Mino radish) of *R. sativus* increased significantly (*P* < 0.05) by 86, 26, and 72%, respectively, in the roots and also increased by 76, 106, and 38% in the leaves in 40 day radish, compared to the untreated plants. Further, the results indicate that the exogenous application of KNO_3_ reduced the MDA, H_2_O_2_, and EL (%) contents by 34, 06, and 51%, respectively, in the roots and also increased by 46, 73, and 20% in 40 day radish compared to the KNO_3_-treated and untreated plants under salt stress. Additionally, compared to plants cultivated without the external application of KNO_3_, oxidative stress indicators diminish with the external application of KNO_3_ in the soil.

### 3.3 Nonenzymatic Antioxidants

**Figure 4:**
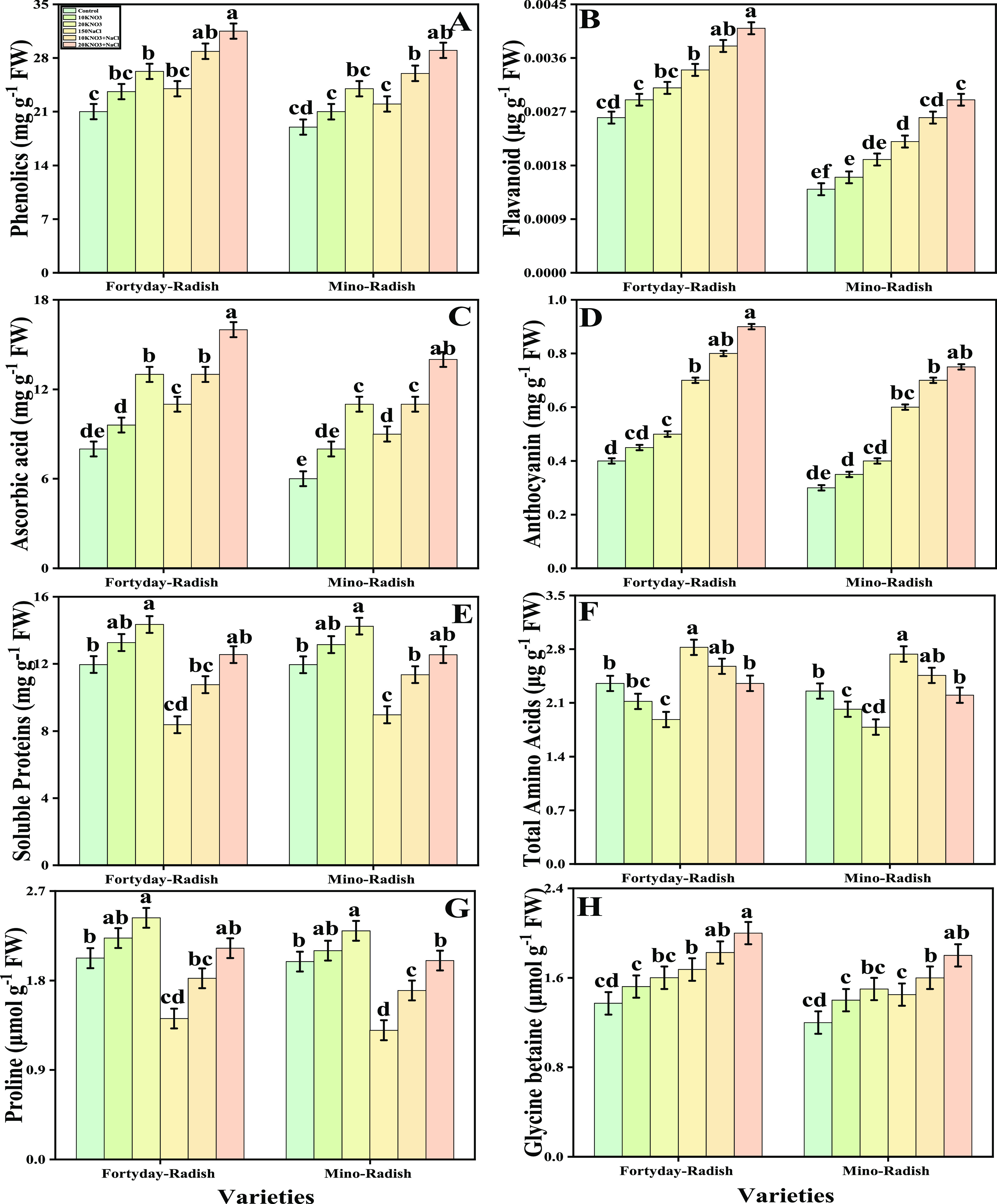
Effect of exogenous potassium nitrate (10 and 20 mM) on phenolic (A), flavonoid (B), ascorbic acid (C), anthocyanin (D), soluble proteins (E), total amino acids (F), proline (G), and glycine betaine (H) of *R. sativus* cultivated under salt stress (150 μM). Bars sharing different letter(s) for each parameter are significantly different from each other according to Duncan’s multiple range test (*P* < 0.05). All of the data represented are the average of four replications (*n* = 4). Error bars represent the standard deviation (SD) of four replicates.

Figure 4 represents the evidence for the presence of nonenzymatic antioxidant compounds such as phenolic, flavonoids, ascorbic acid, and anthocyanin in the two *R. sativus* cultivars (40 day radish and Mino radish) that were grown under salt stress and subjected to exogenous potassium nitrate at different concentrations. The graphical representation elucidates that the contents of phenolic, flavonoids, ascorbic acid, and anthocyanin in the two varieties (40 day radish and Mino radish) of *R. sativus* increased with the exogenous application of KNO_3_ by 41, 43, 24, and 37%, respectively, in the 40 day radish grown under the controlled treatments. More evidence demonstrates that the exogenous KNO_3_ application increased nonenzymatic antioxidant components in 40 day radish in contrast to Mino radish both under control and salt stress conditions. Phenolic, flavonoids, ascorbic acid, and anthocyanin are a few of these substances. Additionally, plants grown with the exogenous addition of KNO_3_ increased the contents of phenolic, flavonoids, ascorbic acid, and anthocyanin in the two cultivars of *R. sativus* (40 day radish and Mino radish) than plants grown without it. The soluble proteins, total amino acids, proline contents, and glycine betaine contents in two varieties (40 day radish and Mino radish) of *R. sativus* with the exogenous application of KNO_3_ under salt stress are also presented in Figure 4. The graphical representation shows that the soluble proteins, total amino acids, proline contents, and glycine betaine contents were decreased by 26, 51, 43, and 50% in 40 day radish, while decreased by 22, 37, 21, and 46% in Mino radish by KNO_3_-untreated plants. Furthermore, compared to plants cultivated without the external application of KNO_3_, the nonenzymatic antioxidants were increased with the external application of KNO_3_ in the soil.

### 3.4 Enzymatic Antioxidants

**Figure 5:**
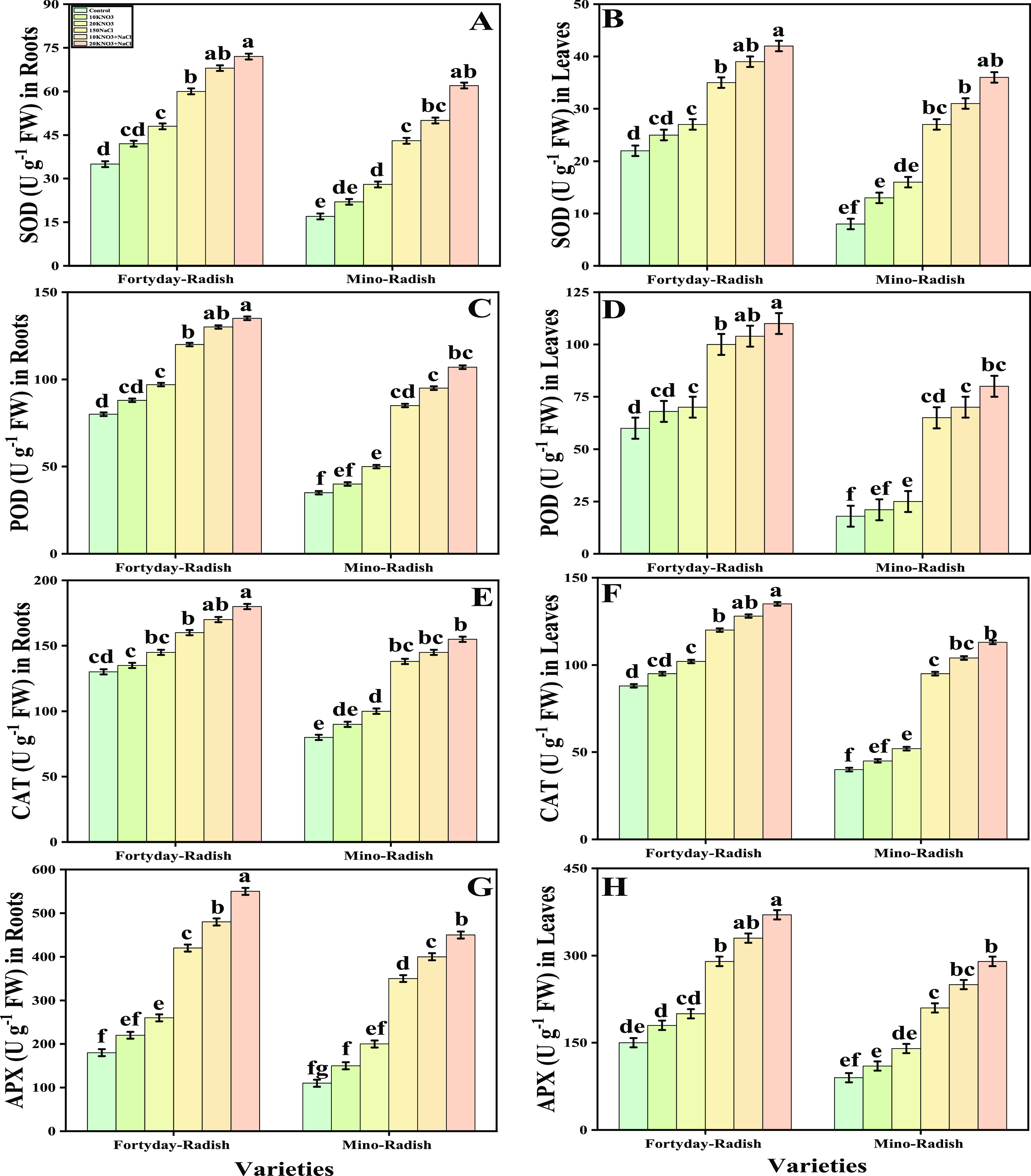
Effect of exogenous potassium nitrate (10 and 20 mM) on SOD activity in roots (A), SOD activity in shoots (B), POD activity in roots (C), POD activity in shoots (D), CAT activity in roots (E), CAT activity in shoots (F), APX activity in roots (G), and APX activity in shoots (H) of *R. sativus* cultivated under salt stress (150 μM). Bars sharing different letter(s) for each parameter are significantly different from each other according to Duncan’s multiple range test (*P* < 0.05). All of the data represented are the average of four replications (*n* = 4). Error bars represent the standard deviation (SD) of four replicates.

Figure 5 represents the data related to the activity of various enzymatic antioxidants, including superoxidase dismutase (SOD), peroxidase (POD), catalase (CAT), and ascorbate peroxidase (APX) in both the roots and leaves of two cultivars (40 day radish and Mino radish) of *R. sativus* by the exogenous application of various levels of KNO_3_ cultivated under salt stress (Figure 5). Results indicated that implementing KNO_3_ exogenously in the soil increased the activities of antioxidants like SOD, CAT, POD, and APX by 64, 24, 36, and 84% in the roots and also increased by 21, 12, 23, and 60% in the leaves of 40 day radish, while also increased by 42, 13, 18, and 60% in the roots and also increased by 13, 14, 16, and 41% in the leaves in Mino radish in comparison to those plants grown without KNO_3_. However, the exogenous application of KNO_3_ increased the antioxidant activities (SOD, POD, CAT, and APX) in the roots and leaves of 40 day radish substantially more than in Mino radish under both control and salt stress conditions. Additionally, compared to plants cultivated without the external application of KNO_3_, the activities of several enzymatic antioxidants increase with the external application of KNO_3_ in the soil.

### 3.5 Nutrient Uptake

**Figure 6:**
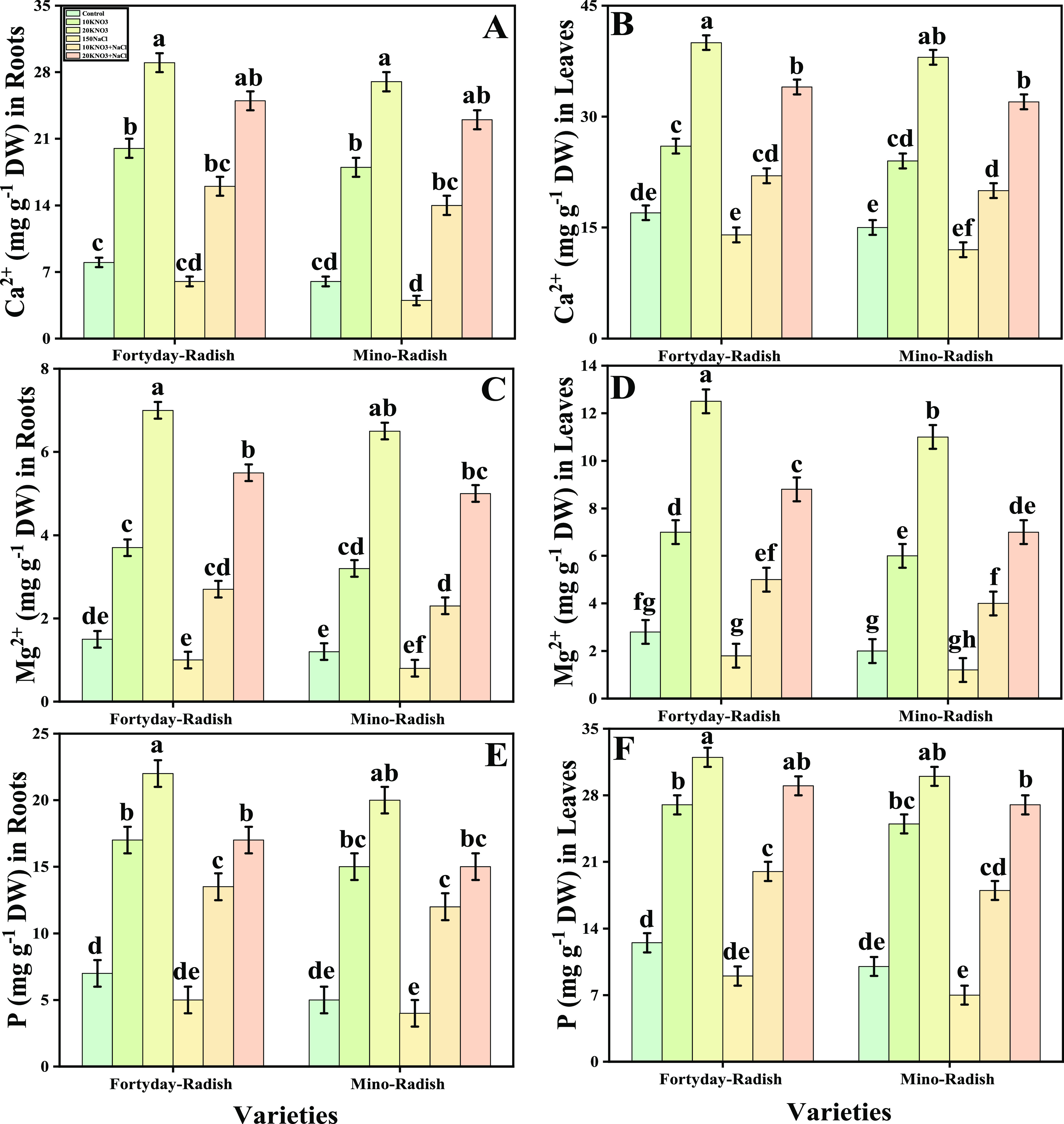
Effect of exogenous potassium nitrate (10 and 20 mM) on calcium contents in roots (A), calcium contents in leaves (B), magnesium contents in roots (C), magnesium contents in leaves (D), phosphorus contents in roots (E), and phosphorus contents in leaves (F) of *R. sativus* cultivated under salt stress (150 μM). Bars sharing different letter(s) for each parameter are significantly different from each other according to Duncan’s multiple range test (*P* < 0.05). All of the data represented are the average of four replications (*n* = 4). Error bars represent the standard deviation (SD) of four replicates.

Figure 6 represents the data related to vital nutrients, i.e., calcium (Ca^2+^), magnesium (Mg^2+^), and phosphorus (P) in both organs (roots and shoots) of both studied cultivars (40 day radish and Mino radish) of *R. sativus* by the foliar application of various levels of KNO_3_ grown under salinity. The graphical representation shows that the calcium (Ca^2+^), magnesium (Mg^2+^), and phosphorus (P) levels decreased by 26, 14, and 26%, respectively, in the roots and also decreased by 24, 10, and 29% in the shoots in 40 day radish, while decreased by 39, 16, and 21% in the roots and also decreased by 24, 26, and 14% in the shoots in Mino radish in both leaves and roots of both the cultivars (40 day radish and Mino radish) of *R. sativus* decreased in salt stress without the exogenous application of various levels of KNO_3_. Further results show that calcium (Ca^2+^), magnesium (Mg^2+^), and phosphorus (P) in plant parts of two cultivars (40 day radish and Mino radish) of *R. sativus* increased by the exogenous application of KNO_3_ under both control and salt stress conditions. Additionally, the findings indicate that the exogenous KNO_3_ application increased the quantity of nutrient uptake in both roots and leaves of both types in contrast to the untreated plants.

### 3.6 Relationship

**Figure 7:**
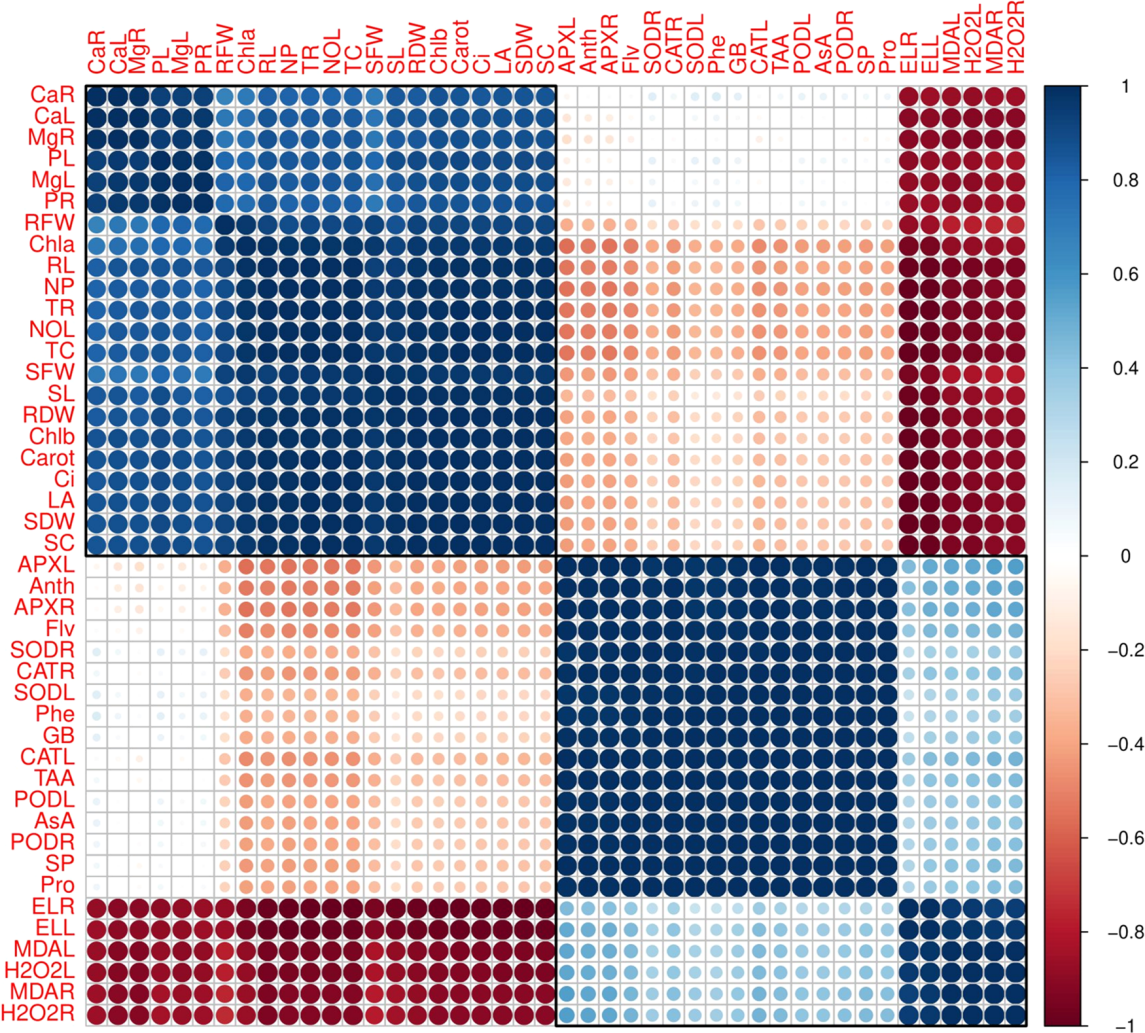
Correlation between different morphological and physiological traits of *R. sativus* studied in this experiment. The abbreviations are as follows: CaR (calcium concentration in the roots), CaL (calcium concentration in the leaves), MgR (magnesium concentration in the roots), PL (potassium concentration in the leaves), MgL (magnesium concentration in the leaves), PR (potassium concentration in the roots), RFW (root fresh weight), Chla (chlorophyll-a content), RL (root length), NP (net photosynthesis), TR (transpiration rate), NOL (number of leaves), TC (total chlorophyll content), SFW (shoot fresh weight), SL (shoot length), RDW (root dry weight), chlb (chlorophyll-b content), carot (carotenoid content), Ci (intercellular CO_2_), LA (leaf area), SDW (shoot dry weight), SC (stomatal conductance), APXL (ascorbate peroxidase activity in the leaves), Anth (anthocyanin content), APXR (ascorbate peroxidase activity in the roots), Flv (flavonoid content), SODR (superoxidase dismutase activity in the roots), CATR (catalase activity in the roots), SODL (superoxidase dismutase activity in the leaves), Phe (phenolic content), GB (glycine betaine content), CATL (catalase activity in the leaves), TAA (total amino acid content), PODL (peroxidase activity in the leaves), AsA (ascorbic acid content), PODR (peroxidase activity in the roots), SP (soluble protein content), Pro (proline content), ELR (electrolyte leakage in the roots), ELL (electrolyte leakage in the leaves), MDAL (malondialdehyde content in the leaves), H_2_O_2_L (hydrogen peroxide content in the leaves), MDAR (malondialdehyde content in the roots), and H_2_O_2_R (hydrogen peroxide content in the roots).

The Pearson correlation analysis was carried out to quantify the relationship between different studied parameters of *R. sativus* grown in saline soil with or without the application of potassium nitrate (Figure 7). Although both varieties showed the same trend, we constructed only one graph (histogram-correlation analysis) of 40 day radish. Calcium concentration in the roots was positively correlated with calcium concentration in the leaves, magnesium concentration in the roots, potassium concentration in the leaves, magnesium concentration in the leaves, potassium concentration in the roots, root fresh weight, chlorophyll-a content, root length, net photosynthesis, transpiration rate, number of leaves, total chlorophyll content, shoot fresh weight, shoot length, root dry weight, chlorophyll-b content, carotenoid content, intercellular CO_2_, leaf area, shoot dry weight, and stomatal conductance, while moderate relationship with ascorbate peroxidase activity in the leaves, anthocyanin content, ascorbate peroxidase activity in the roots, flavonoid content, superoxidase dismutase activity in the roots, catalase activity in the roots, superoxidase dismutase activity in the leaves, phenolic content, glycine betaine content, catalase activity in the leaves, total amino acid content, peroxidase activity in the leaves, ascorbic acid content, peroxidase activity in the roots, soluble protein content, proline content and negative correlation with electrolyte leakage in the roots, electrolyte leakage in the leaves, malondialdehyde content in the leaves, hydrogen peroxide content in the leaves, malondialdehyde content in the roots, and hydrogen peroxide content in the roots. This correlation depicted a close connection between different morphophysiobiochemical attributes of *R. sativus* grown in various concentrations of salinity with or without the application of potassium nitrate.

## 4 Discussion

Plants experience a variety of biotic and abiotic stresses throughout their lives,^34^ including pathogen infestations, water shortages,^35,36^ salts^37,38^ and heavy metals,^39−45^ nutrient imbalance,^46^ and light disturbance, all of which can significantly reduce the yield of many crops around the world.^47−51^ Among them, salinity stress is one of the major constraints that caused substantial damage during plant growth and development that ultimately resulted in reduced crop yields.^5,6^ Under saline stress environments, the higher accumulation of highly soluble salts resulted in altered plants’ physiological and biochemical mechanisms, contributing to heavy yield losses,^52^ whereas the severity of soil salinization is heavily dependent on the soil composition and several environmental factors, i.e., light and plant species. The common responses of plants during saline stress include the inhibition of plant growth, disruption of photosynthetic machinery, alterations in structural composition, exclusion of ions, osmotic readjustments, and modifications in nutrient imbalances. According to the other earlier published studies, lower fresh and dry weight, inhibited photosynthetic rates, and declined absorption of essential nutrients are the common symptoms of saline stress persistence in plants.^52−54^ In accordance with these studies, we also found that the saline stress stunted the plant growth by reducing the photosynthetic rate and absorption of mineral nutrients. Higher oxidation of lipids and proteins and a decrease in photosynthetic pigments are the main reasons that altered the cellular redox status of plants during salt-induced oxidative stress, leading to severe cellular damage in plants.^55^ Nevertheless, the soil salinization imposing drastic effects on plants depends on the total length of the growing cycle, the amount and length of the salinity persistence, and the plant species.

Higher lipid peroxidation is a common phenomenon under salt stress caused by membrane injury, which leads to the production of a number of free oxygen radicals that ultimately disturbed the plants’ functioning and hence the metabolism.^56^ Under salt-induced oxidative stress, the excessive synthesis of ROS is controlled either by ^–^OH and O_2_^–^ or by molecular oxygen excitation (O_2_) to form singlet oxygen.^57,58^ The plants then abruptly activate their antioxidant potential system, which is principally regulated by SOD, POD, CAT, and APX antioxidant enzymes, in response to the excessive ROS production in order to scavenge the ROS and maintain the redox equilibrium, hence boosting plant development.^59−61^ In addition to enzyme antioxidants, nonenzymatic antioxidants including proline, phenolic, and flavonoids work as secondary metabolites to prevent oxidative stress from salt from causing oxidative damage.^62−64^ Similarly in the current study, the elevated levels of oxidative stress biomarkers were noticed in both *Raphanus sativus* L. genotypes, which were then reduced by the stimulation of an antioxidant potential system governed by both enzymatic and nonenzymatic antioxidants. Earlier reports also revealed that the higher activities of antioxidants were observed following saline stress in *H. vulgare*(65)*Cucumis sativus*,^66^ and *Vicia faba.*(67)

Appropriate ion uptake is not only crucial for plant development under normal conditions but also very important during salt-induced oxidative stress conditions since it directly disturbed the cellular redox status by disrupting the ion homeostasis.^68^ Saline stress caused osmotic stress in plants, contributing to the excessive production of soluble salts endogenously that lead to a reduced uptake of essential nutrients.^69,70^ During stressful conditions, the main reasons for nutritional disorders are the availability, absorption or uptake, and distribution of nutrients within the plant.^71^ In saline soils, the obtainability of essential micronutrients mainly depends on the solubility of the micronutrients, occurrence of the binding sites on the surfaces of organic and inorganic particles, soil solution pH and redox potential, and the type of plant species.^66,67^ In the current report, the lower uptake of essential nutrients (Mg^2+^, Ca^2+^, and P) was displayed by both root and shoot of the *Raphanus sativus* L., which were exposed to saline stress as compared nontreated plants. The reason behind this phenomenon is the higher accumulation of NaCl endogenously, as reported by other studies, too.^72,73^

The major source of nitrogen taken up by the plants is nitrate that translocates to the aerial parts and stores in the vacuole and assimilates into reduced nitrogen products.^74^ This assimilation is highly interdependent and leads to a higher synthesis of amino acids and proteins.^75^ The nitrate assimilation heavily depends on its availability and its translocation factors, e.g., light and soil composition.^76−78^ Efficient nitrate uptake and its translocation can be impactful for the mitigation of salt-inducted drastic effects on the nutritional profile of the plants. It has been found that the exogenously applied NO_3_^-^ enhanced the nitrate content in plants grown under saline conditions, thereby improving its tolerance against saline stress to a greater extent.^79,80^ Further, it was noticed that the exogenous application of KNO_3_ significantly stimulated the nitrate content found in ryegrass leaves, saline stress,^81^ and protein and NR contents in tomato and maize.^82,83^ Similarly, the foliar application of KNO_3_ significantly improved the plant’s morphophysiological characteristics under both normal and saline conditions.^84^ In the current investigation, the minimizing effects of the activity of nitrate reductase under saline stress were offset by the exogenous application of mineral. Previously, improved seed germination was observed in grass species by the foliar application of KNO_3_ under saline stress conditions. K+ /Na+ ratio has been proposed as a useful measure for salinity tolerance in wheat plants because K+ is involved in a variety of plant responses.^85^ However, excessive ROS production brought on by salinity frequently results in lipid peroxidation and causes K+ leakage from cells by activating K+ efflux channels.^86,87^ The plasma membrane (PM) H+-ATPase is disturbed when K+ is replaced by Na+ in plant cells due to the elevated Na+/K+ ratio caused by the Na+-induced toxicity under saline stress conditions. Additionally, it was discovered that adding K+ to plants under drought and salinity stress reduced Na+ and increased K+.^88^ Although this study offers new knowledge in the KNO_3_-induced improvement in plant growth and nutrient uptake of *Raphanus sativus* L. plants under saline stress, there is still a wide gap of knowledge to be filled by investigating its ameliorative effects against saline stress at the genetic and molecular levels.

## 5 Conclusions

The current study revealed that the saline stress substantially hampered the growth and development of *Raphanus sativus* L. genotypes due to an increment in levels of oxidative stress indicators, which led to a disturbed defense system of the plants and hence the damaged nutritional profile was noticed. However, better-quality plant growth was seen following the KNO_3_ exogenous application, which was due to the lowering down of the ROS, which resulted in an improved defense system, yielding an improved nutritional profile in both *R. sativus* L. genotypes under normal and salt stress conditions. These outcomes supported the ameliorative effects of KNO_3_ on radish growth, ultimately conferring salt tolerance in *R. sativus* L.

## Data Availability

The data presented in this study are obtained during the original experiment.
